# The Antiviral Efficacy of HIV-Specific CD8^+^ T-Cells to a Conserved Epitope Is Heavily Dependent on the Infecting HIV-1 Isolate

**DOI:** 10.1371/journal.ppat.1001341

**Published:** 2011-05-12

**Authors:** Srinika R. F. Ranasinghe, Holger B. Kramer, Cynthia Wright, Benedikt M. Kessler, Katalin di Gleria, Yonghong Zhang, Geraldine M. Gillespie, Marie-Eve Blais, Abigail Culshaw, Tica Pichulik, Alison Simmons, Sarah L. Rowland-Jones, Andrew J. McMichael, Tao Dong

**Affiliations:** 1 Medical Research Council Human Immunology Unit, Weatherall Institute of Molecular Medicine, University of Oxford, John Radcliffe Hospital, Oxford, United Kingdom; 2 Henry Wellcome Building of Molecular Physiology, Nuffield Department of Clinical Medicine, University of Oxford, Oxford, United Kingdom; 3 BeiJing You'An Hospital, BeiJing Capital University, BeiJing, China; NIH/NIAID, United States of America

## Abstract

A major challenge to developing a successful HIV vaccine is the vast diversity of viral sequences, yet it is generally assumed that an epitope conserved between different strains will be recognised by responding T-cells. We examined whether an invariant HLA-B8 restricted Nef_90–97_ epitope FL8 shared between five high titre viruses and eight recombinant vaccinia viruses expressing Nef from different viral isolates (clades A–H) could activate antiviral activity in FL8-specific cytotoxic T-lymphocytes (CTL). Surprisingly, despite epitope conservation, we found that CTL antiviral efficacy is dependent on the infecting viral isolate. Only 23% of Nef proteins, expressed by HIV-1 isolates or as recombinant vaccinia-Nef, were optimally recognised by CTL. Recognition of the HIV-1 isolates by CTL was independent of clade-grouping but correlated with virus-specific polymorphisms in the epitope flanking region, which altered immunoproteasomal cleavage resulting in enhanced or impaired epitope generation. The finding that the majority of virus isolates failed to present this conserved epitope highlights the importance of viral variance in CTL epitope flanking regions on the efficiency of antigen processing, which has been considerably underestimated previously. This has important implications for future vaccine design strategies since efficient presentation of conserved viral epitopes is necessary to promote enhanced anti-viral immune responses.

## Author Summary

One of the greatest challenges to developing an effective HIV vaccine is the ability of HIV to rapidly alter its viral sequence. Such variation in viral sequence enables the virus to frequently evade recognition by the host immune system. To counteract this problem, there has been increasing interest in developing HIV vaccines that target T-cell responses to the regions of the virus that are highly conserved between strains of HIV. However, previous studies have focused on identifying amino acid variation predominantly within a single viral isolate, or have focused on classical within-epitope escape mutation. Our study assessed T-cell recognition of a conserved epitope shared by a total of 13 HIV strains. Strikingly, we show that only a small proportion of the viral strains were effectively recognised and targeted by the T-cells. In contrast, differences in amino acid sequence in the region flanking the epitope impaired the intracellular processing and presentation of epitope in the majority of HIV strains tested. Thus, our findings highlight that a large proportion of HIV strains may evade epitope-specific T-cell recognition despite absolute epitope conservation. This has important implications for both vaccine design and evaluation of vaccine efficacy.

## Introduction

One of the greatest challenges in developing an effective T-cell based vaccine against HIV-1 is its high genetic variability [1]. Group M HIV-1 has expanded globally into 15 major clades, sub-clades and several interclade circulating recombinant forms. These continually evolving HIV-1 clades differ by over 30% in envelope amino acid sequences and viral isolates within the same clade may also differ by up to 15% [2]. The high rate of mutation from error-prone reverse transcription combined with replicative ability enables HIV-1 to adapt rapidly to immune and drug pressure, with the generation of multiple genetically distinct quasispecies within infected individuals. HIV-1 vaccines must overcome these obstacles to induce protective immunity against heterologous viral variants [3].

A critical component of HIV-1 control during the acute phase is the cytotoxic T-lymphocyte (CTL) response [4], [5]. Therefore, many current vaccine strategies focus on identifying immunogens that elicit effective T-cell immunity against a diverse range of viral variants and characterising HIV-1 specific CTL responses in order to define the immune correlates of protection [5]. To counteract antigenic diversity, there is an increasing interest in developing HIV vaccines which elicit CTL responses to conserved epitopes, centralised sequences or immunogenic regions of high inter-clade homology [6], [7], [8], [9], [10]. Currently, the interferon gamma (IFNγ) producing ELISpot assay is frequently used to quantify the breadth and magnitude of CTL responses [11], using peptides matched to consensus virus sequence or occasionally to autologous infecting virus sequence [12] [13]. Most data show that CTL recognition of epitope peptides is very sensitive to any change in the epitope peptide [13], [14]. Thus the HLA type of the patient imprints changes on the sequence of the infecting virus, generally thought to be within the epitopes that have stimulated CTL responses [15], [16]. However, while CTL may efficiently recognise exogenously loaded synthetic peptide matched to HIV-1 clade variants, it has been found that this does not necessarily correlate with CTL antiviral activity against HIV-1 infected cells displaying endogenously derived peptides [17]; for example, the artificial peptides may be added at non-physiological concentrations. Therefore, conventional peptide-based assays may over-estimate the ability of CTL to cross-recognise variant epitopes [18]. The use of exogenous synthetic peptides to quantify CTL responses may also fail to detect differences in the antiviral efficacy of CD8 T-cells that reflect variation in antigen processing efficiency within HIV-infected cells [19], [20]. Remarkably, whilst much research has focused on recognition of exogenously added peptide epitopes, CTL recognition of virus-infected cells has been examined relatively rarely, and there has been no analysis of CTL recognition of invariant epitopes shared by diverse viral isolates and clades.

The present study arose from the observation that CD8+ T cells specific for the highly conserved HLA B8 restricted Nef epitope FLKEKGGL (FL8) failed to recognize HLA B8 positive cells infected with several HIV-1 isolates. Previously it has been shown that escape mutations can occur in the epitope flanking regions through impaired processing and presentation [21], [22], [23], [24], [25], [26], [27], however, such studies have focused predominantly on a single viral isolate, mostly in circulating virus rather than selected viral strains in individual patients, or focused on classical escape mutation [28]. Therefore a range of HIV-1 isolates and vaccinia viruses expressing different Nef proteins, each of which shared this conserved epitope, were used to test responses from a set of CTL clones isolated from HLA B8+ patients. Overall, we evaluated CTL recognition and antiviral efficacy induced by a total of thirteen viral isolates containing the same conserved epitope. Surprisingly, only a small proportion (23%) of these HIV-1 isolates induced optimal CTL recognition and antiviral efficacy. We found that variations in the flanking region had a profound effect on the presentation of this epitope, and viral isolates within the same HIV-1 clade were differentially recognized by FL8-specific CTL clones. Furthermore, we identified a phenylalanine motif in the FL8-epitope flanking regions of four HIV-1 isolates that led to an altered pattern of cleavage by the immunoproteasome that correlated with loss of CTL recognition.

## Results

### CTL recognition of exogenous synthetic peptide does not correlate with CTL antiviral activity against endogenous peptide presented *de novo* on the surface of HIV-infected cells

**Figure 1:**
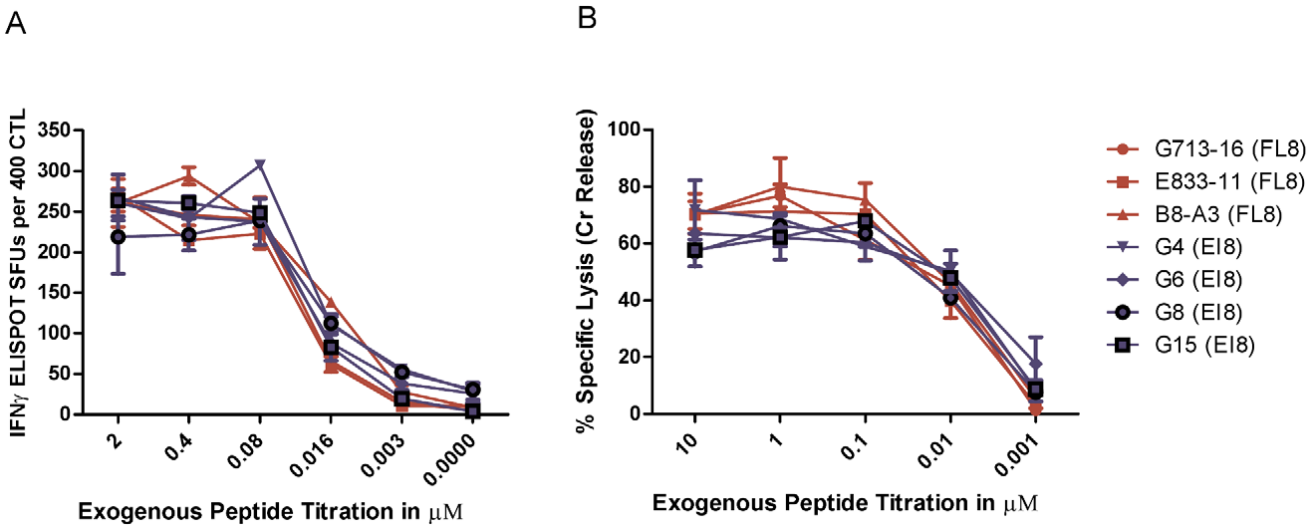
Nef FL8-specific and Gag EI8-specific CTL clones demonstrate similar functional avidity. Synthetic FL8 and EI8 peptides were exogenously titrated on uninfected CD4+ Tcell line C8166 with their respective panel of FL8- and EI8-specific clones in a conventional IFNγ ELISPOT at an E∶T ratio of 400∶20000 (A) and 51Cr release assay at an E∶T ratio of 40000∶5000 (B) per well. FL8- specific clones are shown in red and control EI8-specific clones are shown in blue. The standard error results represent three independent experiments.

In conventional IFNγ ELISPOT and chromium release assays, we assessed three Nef FL8-specific CTL clones and four control Gag EI8-specific CTL clones for their recognition of HLA-B8^+^ matched C8166 target cells pulsed with peptides at different concentrations to measure functional avidity (the peptide concentration that gives 50% maximum effect). The FL8-specific and EI8-specific CTL clones had comparable levels of functional avidity, measured by IFNγ release (Figure 1A) and in a lytic assay (Figure 1B). In addition, there were no significant differences (p>0.05) observed between FL8- and EI8- specific responses in the lysis assay at all peptide concentrations tested. This suggested that both peptides would be equally recognised in HLA-B8 target cells infected with HIV-1.

We then compared CTL recognition of synthetic Nef FL8 peptide pulsed exogenously onto the surface of uninfected C8166 targets with CTL antiviral efficacy against endogenously derived FL8 epitope presented on the cell surface of HIV-1_HXB2_ infected C8166 targets. Surprisingly, FL8-specific CTL did not recognise or mount an antiviral response against the virus-infected target cells.

**Figure 2:**
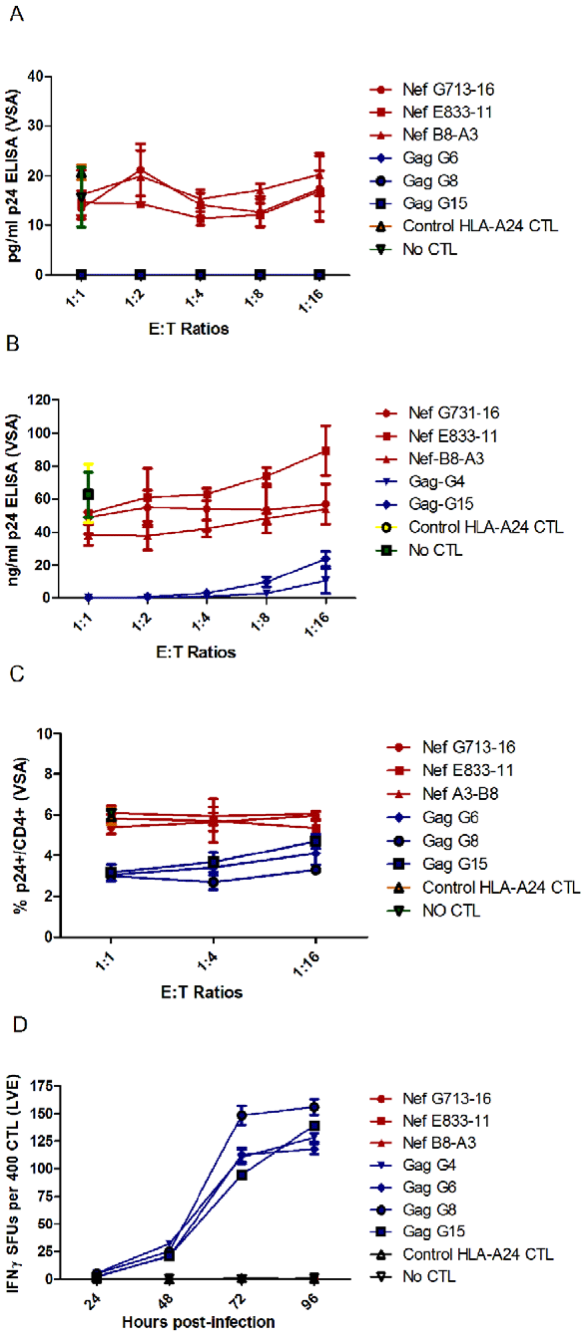
Nef FL8-specific CTL clones show negligible anti-viral efficacy to HIV-1_HXB2_ infected cells. In the Viral Suppression Assay (VSA), the CD4+ T-cell line C8166 was infected separately with low titre (A) and high titre (B) HIV-1_HXB2_ and co-cultured with a panel of FL8- and EI8-specific clones at several E∶T ratios for 4 days and the viral supernatant was subsequently assayed by p24 ELISA. The low-titre infected co-culture was also assayed by intracellular p24 staining (C). In the Live Virus ELISPOT (LVE) assay, C8166 was infected with HIV-1_HXB2_ for a period of 24, 48, 72 and 96 hours post-infection. At each respective time point, infected cells expressing endogenously derived epitopes were co-cultured with their respective panel of FL8- and EI8- specific CTL clones on a pre-coated IFNγ ELISPOT for 6 hours at an E∶T ratio of 400∶20000 (D). FL8- specific clones are shown in red and control EI8-specific clones are shown in blue. The standard error represents each clone tested in triplicate for every respective E∶T ratio or time point and is representative of three independently performed assays.

In a Viral Suppression Assay (VSA), the panel of FL8- and EI8- specific CTL clones were co-cultured for four days with C8166 target cells infected with HIV-1_HXB2_ at five E∶T ratios, after which suppression of viral replication in the supernatant was quantified by measuring the level of Gag p24 by ELISA; and suppression of HIV-infected targets (via CTL lysis or non-cytolytic inhibition) was measured using intracellular p24 staining. Despite the similar functional avidity in peptide based assays, at a low infectious titre of virus, EI8-specific CTL exhibited superior suppression of viral replication compared to FL8-specific CTL when p24 was measured in supernatant by ELISA (Figure 2A). At all E∶T ratios tested, co-culture with EI8-specific CTL resulted in undetectable levels of p24 Gag (below the ELISA threshold of 10 pg/ml) whilst viral suppression by FL8-specific CTL was negligible when compared to wells of virus-infected targets in the absence of CTL. Analysis of suppression at a high infectious titre showed similar results, with negligible suppression of virus-infected cells by FL8-specific T-cells at all E∶T ratios tested (ranging from 37–89 ng/ml with a mean p24 of 62 ng/ml in the absence of CTL) (Figure 2B). Subsequent analysis of the p24 stained cells showed a similar pattern (Figure 2C). At the 1∶1 and 1∶4 ratios, the control EI8 specific CTL clones demonstrated effective inhibition of viral replication, although they were unable completely to abrogate infection, whilst FL8-specific CTL did not differ from the controls in their suppression of viral infection at all three ratios tested. 2-way ANOVA with bonferroni post-test confirmed a statistically significant difference (p<0.01 or p<0.001) between Nef-specific and Gag-specific clones at each E∶T ratio in the ELISA.

We also developed a Live Virus Elispot (LVE) assay to assess IFNγ release by CTL exposed to HIV-infected target cells, as a marker of CTL antiviral activity over time. C8166 target cells were infected with HIV-1_HXB2_ and incubated for a period of 24, 48, 72 and 96 hours at 37°C. At each respective time point, the HIV-infected cells were co-cultured with HLA-matched FL8- and EI8-specific CTL clones on a pre-coated IFNγ ELISpot plate. Again, FL8-specific CTL did not mount an IFNγ response at any of the time points tested (Figure 2D). In contrast, the EI8 epitope was recognised by the EI8-specific CTL clones, which generated a detectable IFNγ response that was significantly different (p<0.001) between FL8- and EI8-specific clones at 48, 72 and 96 hours post-infection in a 2-way ANOVA with bonferroni post-test.

The three Nef FL8-specific clones were generated from three separate HLA-B8^+^ long-term non-progressors (LTNP) and control Gag EI8-specific clones were generated from one HLA-B8^+^ LTNP patient. The use of this pre-screened panel of T-cell clones removes the complexity of different TCR affinities, and thus variation in TCR/pMHC interactions, of polyclonal T-cell responses.

### Significant variation in CTL antiviral efficacy to a conserved Nef epitope shared between HIV isolates and clades

To investigate whether CTL recognition and antiviral activity to a conserved epitope may depend on the infecting HIV-1 isolate, we chose five high titre HIV viruses (three clade B laboratory strains and two clade A isolates) that share the invariant Nef FL8 epitope for testing via *in vitro* Viral Suppression Assays (VSA) and Live Virus ELISPOTS (LVE). FL8 is an immunodominant epitope that is highly conserved amongst HIV-1 Group M isolates in the Los Alamos National Laboratory (LANL) HIV sequence database. Proviral DNA for each virus was isolated from control wells containing infected C8166 targets in the absence of CTL, PCR amplified and sequenced to confirm the presence of the invariant FL8 epitope. The results from both assays and viral sequencing are summarised in Table 1.

**Table 1:** Despite the identical FL8 epitope, only a small proportion (23%) of these HIV-1 isolates induced optimal CTL recognition and antiviral efficacy.

High Titre HIV Isolates	Clade	Viral Sequence for FL8 and flanking region	Viral Suppression Assay	Live Virus ELISPOT
HIV-1_92UG029_	A	**VGFPVRPQVPLRPMTYKGAVDLSHFLKEKGGLDGLIYSRKRQEILDLWVYNTQGYF**	++	++
HIV-1_93RW024_	A	**VGFPVRPQVPLRPMTYKAAVDLSHFLKEKGGLEGLIYSRRRKDILDLWVYHTQGFF**	+++	+++
HIV-1_HXB2IIIB_	B	**VGFPVTPQVPLRPMTYKAALDLSHFLKEKGGLDGLIYSQKRQDILDLWVYHTQGYF**	−	−
HIV-1_MN_	B	**VGFPVRPQVPLRPMTYKAALNLSHFLKEKGGLDGLIYSQKRQDILDLWVYHTEGYF**	+++	++
HIV-1_89.6_	B	**VGFPVRPQVPLRPMTYKAALDLSHFLKEKGGLDGLIHSQKRQDILDLWVYHTEGYF**	−	−

rVV-Nef HIV Isolates	Clade	Viral Sequence for FL8 epitope and flanking region	^51^Cr-release Assay	IFNγ ELISPOT
HIV-1_92UG037_	A	**VGFPVRPQVPLRPMTYKAAFDLGFFLKEKGGLDGLIYSKKRQEILDLWVYHTQGYF**	−	−
HIV-1_MN_	B	**VGFPVKPQVPLRPMTYKAALDLSHFLKEKGGLDGLIYSQKRQDILDLWVYHTQGYF**	++	++
HIV-1_96ZM651_	C	**VGFPVRPQVPLRPMTYKAAVDLSFFLKEKGGLEGLIYSKKRQEILDLWVYHTQGFF**	+	+
HIV-1_94UG114_	D	**VGFPVRPQVPLRPMTYKEAVDLSHFLKEKGGLEGLVWSPKRQEILDLWVYHTQGFF**	+++	+++
HIV-1_CM235_	A/E	**VGVPVRPQVPLRPMTYKGAFDLSFFLKEKGGLEGLIYSKRRQEILDLWVYNTQGFF**	−	−
HIV-1_93BR020_	F	**VGFPVRPQVPLRPMTYKGAVDLSHFLKEKGGLEGLIYSKRRQEILDLWVYHTQGYF**	++	++
HIV-1_92NG083_	G	**VGFPVRPQVPLRPMTYKAAFDLSFFLKEKGGLDGLIYSKRRQDILDLWVYNTQGFF**	−	−
HIV-1_90CF056_	H	**VGFPVRPQVPLRPMTYKGAFDLSHFLKEKGGLDGLIYSKQRQDILDLWVYNTQGYF**	++	−

**Figure 3:**
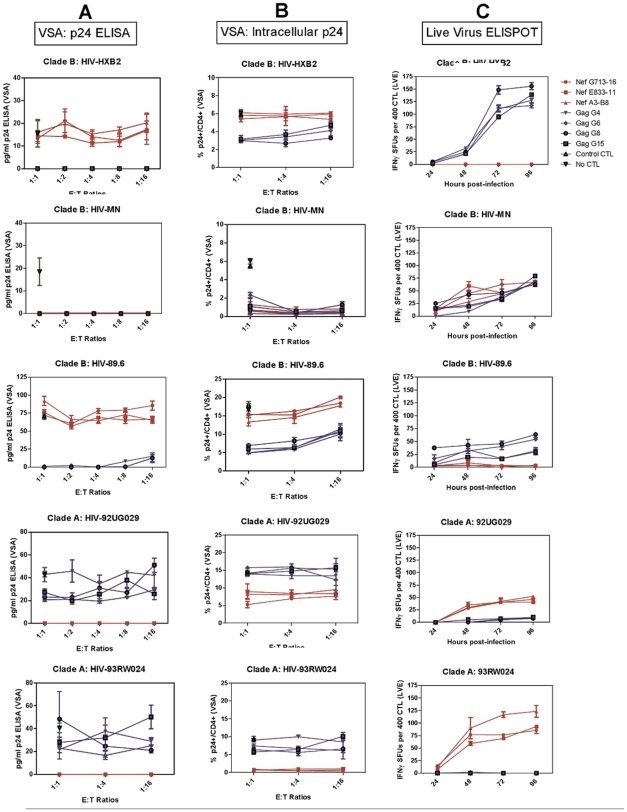
Despite the identical FL8 epitope, only 3/5 viruses tested induced effective FL8-specific antiviral responses. Viral Suppression Assay including p24 ELISA of viral supernatant (A) and intracellular p24 staining of infected co-culture (B), and the Live Virus Elispot (C). FL8- specific CTL responses are shown in red and control EI8-specific CTL responses are shown in blue. Each assay was independently performed at least 3 times for each virus.

**Figure 4:**
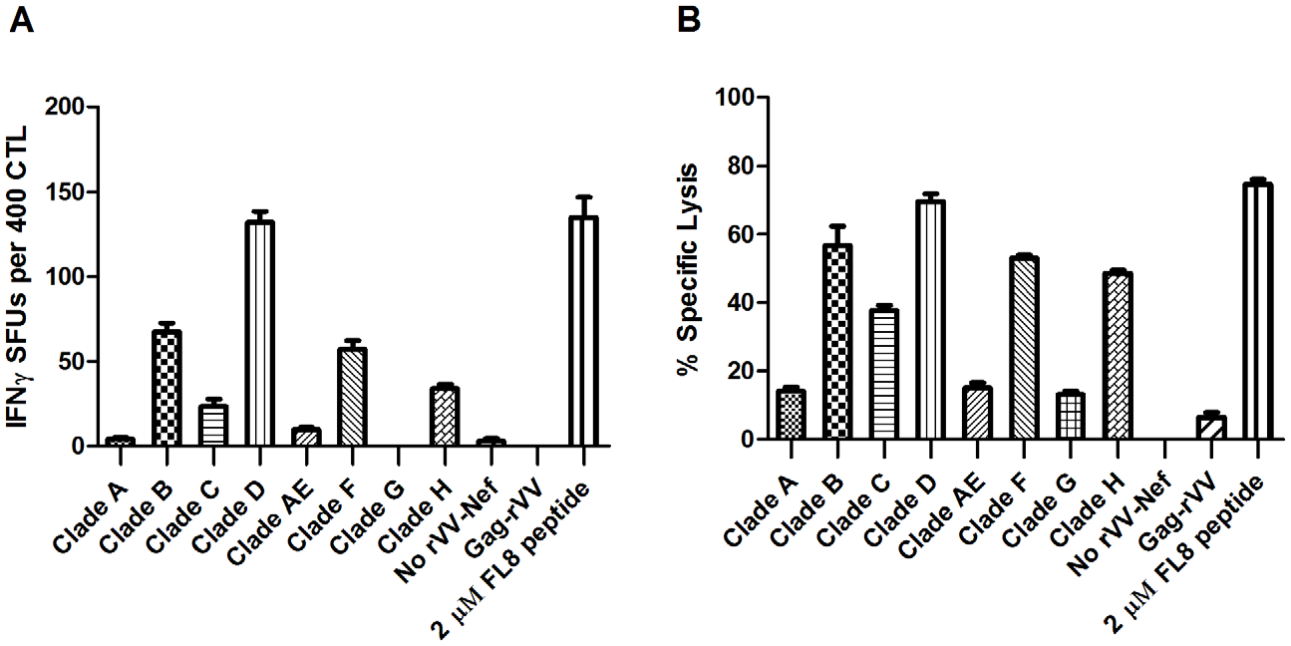
7/8 recombinant vaccinia viruses expressing Nef (rVV-Nef) from different viral isolates show impaired or abolished antiviral activity. A HLA-B8 B-cell line was infected separately with each rVV-Nef and co-cultured with an FL8-specific clone in a IFNγ ELISPOT at an E∶T ratio of 400∶20000 (A) and ^51^Chromium release assay at an E∶T ratio of 40000∶5000 per well (B). The standard error results represent three independent experiments.

The results from the Viral Suppression Assay and Live Virus Elispot in Figure 3 and IFNγ ELISPOT and CTL Lysis assays in Figure 4 are summarised with +++ for optimal, ++ for impaired, + for severely impaired and − for abolished epitope processing and CTL antiviral efficacy.

Data from the viral suppression assay for each virus shows differing FL8-specific CTL antiviral activity for the different virus isolates, despite sharing the conserved FL8 epitope (Figure 3). FL8 specific CTL suppressed both clade A viral isolates HIV-1_92UG029_ and HIV-1_93RW024_, as well as Clade B HIV-1_MN_, to below the threshold of detection (10 pg/ml) in the p24 ELISA (Figure 3C). However, clade B HIV-1_HXB2_ (as characterised before in Figure 2A) and clade B HIV-1_89.6_ were not suppressed by FL8-specific CTL at any of the effector∶target (E∶T) ratios tested, and were not statistically different (p>0.05) when compared to wells of virus-infected cells in the absence of CTL. Similar trends were obtained when analysing the corresponding infected co-cultures from the VSA (Figure 3B). In HIV-1_92RW024_ infected target cells, FL8 specific CTL reduced infection from 9% to <1% whilst a reduction was also observed in HIV-1_92UG029_ infected cells at all three E∶T ratios tested, which were significantly different (p<0.01) from control wells of infected cells in the absence of CTL. The clade B virus HIV-1_MN_ was also efficiently suppressed by FL8 and EI8 CTL. However, FL8-specific CTL exerted no significant antiviral efficacy against HIV-1_HXB2_ or HIV-1_89.6_ infected target cells, suggestive of impaired intracellular FL8 epitope processing and presentation. Similar result was observed while using HIV-1_HXB2_ and MN infected HLA B8+ PBMCs as target cells in VSA (Figure S2).

Differential CTL antiviral activity to the FL8 epitope between viral isolates was also observed in the Live Virus Elispots (LVE) assay (Figure 3C). FL8-specific CTL demonstrated a strong IFNγ response with an average magnitude of 46–101 SFUs at 96 hours post-infection against endogenously presented FL8 peptide by cells infected with clade A isolates HIV-1_92UG029_ and HIV-1_92RW024_ respectively. In contrast, a varied FL8-specific CTL response was observed against clade B infected cells, with a mean of 65 SFUs recorded for HIV-1_MN_, but 0 SFUs at 96 hrs post-infection for both HIV-1_HXB2_ and HIV-1_89.6_. Further LVE studies were conducted at 6 and 12 hours post-infection to assess ‘early’ CTL recognition of endogenous Nef and Gag epitopes but no IFNγ responses were observed.

All high titre viruses were tested in parallel with the same panel of three highly avid FL8-specific clones and four control EI8-specific clones with the same results. All five viruses were CXCR4-tropic, able to replicate efficiently and were pathogenic, as confirmed by syncytia formation with 6–20% of C8166 cells (p24+/CD4+) infected by Day 4 in the absence of CTL. A low MOI was chosen for physiologic relevance to an *in vivo* setting, with <20% infection observed, comparable to other SIV and HIV- based viral suppression assays [21], [29]. However, similar results were also obtained at a higher MOI, so viral titre does not appear to impact upon the patterns observed (as observed in figure 2B). Furthermore, since HIV-1_HXB2, MN_ and _93RW024_ contained a conserved HLA-A24 restricted RW8 Nef epitope, we were also able to utilise CTL clones specific for RW8 to confirm that these viruses were able to present Nef derived peptides to CTL and were not simply Nef-deficient (Figure S1). Interestingly, although there is a general trend towards higher p24 at the lower E∶T ratios with the control gag clones, and with the Nef clones to a lesser extent, overall the E∶T ratio appears to have limited influence in the VSA. This is in accordance with the results from a similar assay [30] and may be attributable to the use of CTL clones that were pre-selected for their high avidity and ability to suppress viral replication at low E∶T ratios (in comparison to polyclonal populations with differing avidity). It should also be noted that our control Gag EI8 clones specific to EIYKRWII were chosen as an internal control as they had similar functional avidity for the clade A intra-epitopic variant DI8 (DIYKRWII) when tested with peptides in the IFNγ ELISPOT and chromium release assays (data not shown). However, these Gag-specific CTL were unable to respond to DI8 targets within the clade A viruses HIV-193RW024 and HIV-192UG029 in Figure 3, due to more efficient immunoproteasomal processing for EI8 than DI8 (data not shown); however they still acted as good internal controls for clade B infections.

Overall, our data from the viral suppression assays and live virus ELISPOTS clearly demonstrate that efficient CTL antiviral activity is heavily dependent on the infecting HIV-1 isolate, which cannot be accurately predicted from epitope sequence conservation alone or by clade-grouping.

### Significant variation in CTL recognition of a conserved FL8 epitope from recombinant vaccinia viruses expressing Nef from eight different viral isolates (clades A–H)

We also infected HLA-B8 B-cells with recombinant vaccinia virus constructs expressing HIV-1 Nef (rVV-Nef) from eight viral isolates, each from a different group M clade (for simplicity abbreviated by their clade reference): HIV-1_92UG037.1_(A), HIV-1_MN_ (B), HIV-1_96ZM651_ (C), HIV-1_94UG114.1_ (D), HIV-1_CM235-32_ (AE), HIV-1_93BR020_ (F), HIV-1_92NG83.2_ (G) and HIV-1_90CF056_ (H). The rVV-Nef expressing cells were co-cultured with FL8-specific CTL in two assays; a ^51^Cr-release assay and IFNγ ELISPOT. The results from both assays and the pre-determined rVV-Nef sequences are summarised in Table 1.

In the ^51^Cr-release assay, striking differences were observed in the level of CTL-induced lysis of cells infected with rVV-Nef from different viral isolates, which could be categorised into three distinct groups based upon their mean percentage lysis. Only in cells infected with rVV-Nef-D was the proportion of lysed cells (70%) comparable to peptide-pulsed targets (75%) indicative of optimal abundance of endogenously derived FL8 for efficient recognition and lysis (Figure 4B). Subsequent analysis confirmed that there was no significant difference between rVVNef-D and the control in an ANOVA (p>0.05). In contrast, infection with viral isolates B, C, F and H resulted in reduced CTL recognition, with only 20–70% lysis compared to the peptide control, whilst no significant CTL killing (>20% lysis) was observed for isolates A, AE and G, suggestive of impaired or abolished FL8 processing and presentation. Concordant results were observed in the IFNγ ELISPOT (Figure 4A). Only isolate D elicited levels of IFNγ release (132 SFCs) similar to the peptide control (135 SFCs). In contrast, infection with B, C, F and H showed intermediate levels of 67, 23, 57 and 34 SFCs respectively and less than 10 SFCs for A, AE and G. In total, seven rVV-Nefs elicited CTL antiviral responses that were significantly different (p<0.01 or p<0.001) from the CTL response to the FL8 peptide control. These data support the isolate-specific nature of CTL antiviral efficacy, despite epitope conservation between viruses.

To ensure similar levels of Nef protein expression in these assays, rVV infection used the same plaque forming unit (pfu) titre and a CTL clone specific to a conserved Nef epitope HLA-A3 QK10 was utilised as an internal control. The strong CTL response to endogenous QK10 peptide presented by rVV-A, AE and G in the chromium release assay confirmed that these rVV efficiently infected the BCL targets and were not Nef-defective (Figure S1).

### HLA-B8 expression is not significantly altered in cells infected with HIV-1 high titre isolates or recombinant vaccinia viruses expressing HIV-1 isolate specific Nef protein

To verify that the striking differences in CTL antiviral activity to a conserved epitope observed in Figure 3 and 4 were not attributable to differing Nef-induced down-regulation of surface MHC class I expression by the various virus isolates [31], [32], we infected target cells with virus and assessed HLA-B8 surface expression using a HLA-B8 specific monoclonal antibody. We compared HLA-B8 expression in HLA-B8^+^ targets infected with our five high titre HIV-1 isolates (48 hours post-infection) and eight rVV-Nef constructs (4 hours post-infection), and also utilised three HLA-B8^−^ negative targets as a negative control.

**Figure 5:**
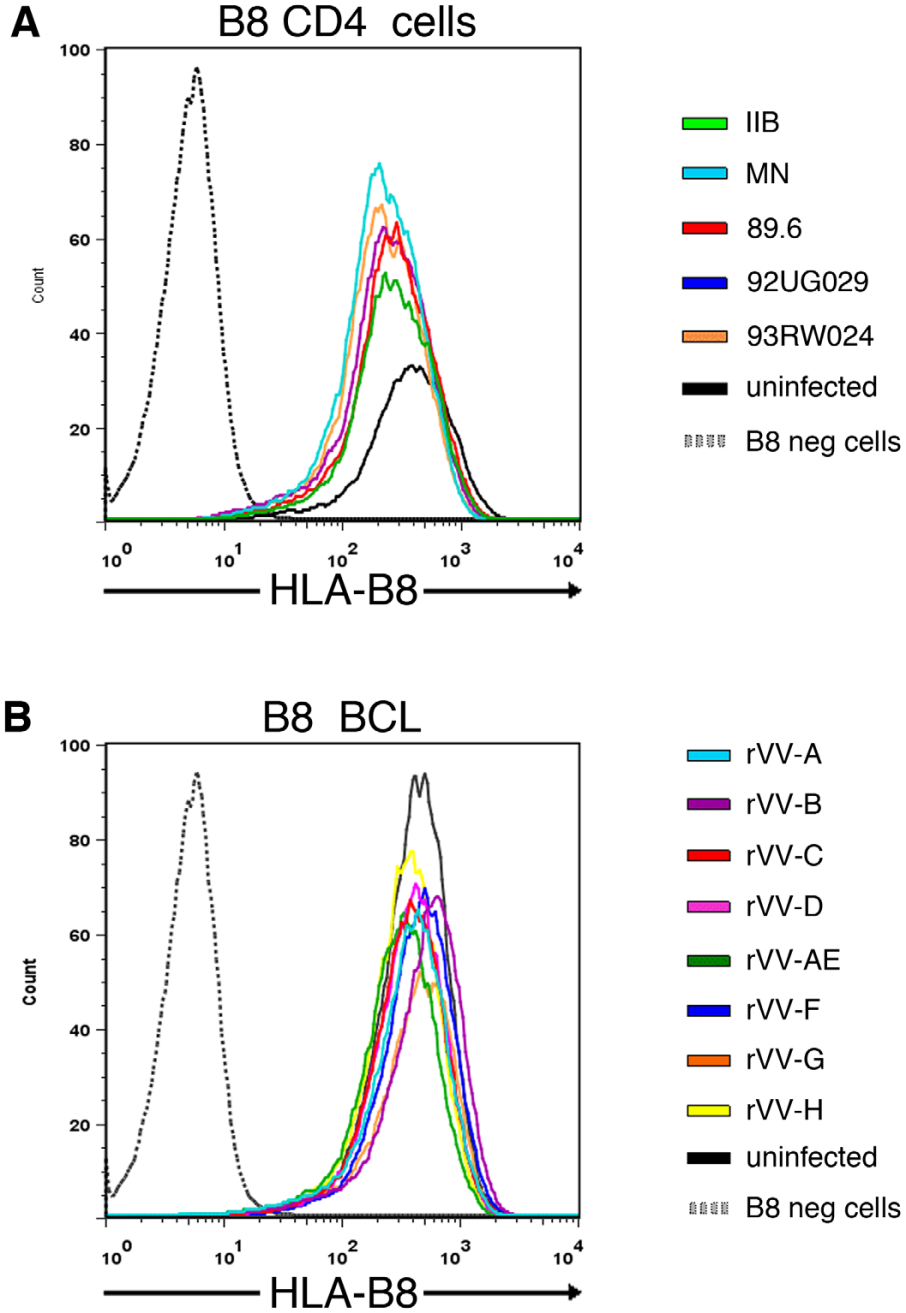
No marked difference in HLA-B8 surface expression between target cells infected by individual HIV isolates or rVV-Nef constructs. C8166 targets were infected with the five high titre viruses for 48 hours post-infection (A) or HLA-B8+ BCL were infected with eight recombinant vaccinia viruses expressing Nef for 4 hours post-infection (B). HLA-B8 surface expression was assessed using a HLA-B8 specific monoclonal antibody via flow cytometry. HLA-B8 uninfected cells are shown in black and HLA-B8- negative cells are shown in grey dashed lines as negative controls.

Our results showed that although down-regulation of HLA-B8 surface expression was observed in the high titre virus infected cells when compared with uninfected or vaccinia-infected targets, there was no marked variation in HLA-B8 between the five high titre HIV-infected target cells (Figure 5A). Furthermore, there was no marked variation in HLA-B8 expression between the eight rVV-infected target cells (Figure 5B). However, we acknowledge that the direct measurement of HLA-B8 expression may vary between experiments. Whilst Lewis et al controlled for this with internal standards [33], Nef-deleted viruses were not available for comparison with our wild-type viruses. Alternatively, we utilised B8-restricted Gag CTL controls in our viral suppression assays and live virus Elispots, since any effect of Nef mediated down-regulation of HLA-B8 ought to apply equally to this epitope and not be restricted to Nef epitopes. The CTL recognition of the Gag epitope was the same for the viruses tested, even when Nef recognition differed markedly. Together, these data therefore demonstrate that Nef-mediated down-regulation of HLA-B8 is not responsible for diminished CTL recognition of the conserved FL8 epitope on cells infected with HIV-1_HXB2_ and _89.6_ isolates, or with rVV-Nef constructs A, AE and G.

### Proteasomes and immunoproteasomes are important in the endogenous generation of the Nef FL8 epitope

From the above results it seemed likely that differences in antigen processing might explain the failure of CTL to recognise cells infected with some of the virus isolates and rVV-Nef. Intracellular peptide processing is a multi-step pathway and amino acid variations within epitopes and their flanking regions can affect any step of this complex cascade [34], which includes proteasomal or immunoproteasomal cleavage, TAP mediated transport to the ER lumen and N-terminal trimming by aminopeptidases. Alternatively, other proteases such as tripeptidyl peptidase II (TPPII) may act independently or in combination with the proteasome to generate epitope precursors. TPPII is known to play a pivotal role in the generation of the immunodominant HLA-A3 Nef QK10 epitope [35], which is located 8 amino acid residues downstream from the HLA-B8 Nef FL8 epitope.

To test the importance of different components of the antigen processing machinery on the generation and presentation of FL8 on the infected-cell surface, we chose two viruses that elicited a dominant FL8 response; high titre HIV-1_92UG029_ and rVV-D (HIV-1_94UG114_). Infected target cells were treated with inhibitors to block proteasome and immunoproteasome (Epoxomicin), aminopeptidase activity (Bestatin) and tripeptidyl-peptidase II activity (AAF-CMK) at appropriate concentrations. Controls included inhibitor treated cells pulsed with peptide (both HIV-infected and uninfected) to ensure that the read out was not altered by inhibitor-induced cell death.

**Figure 6:**
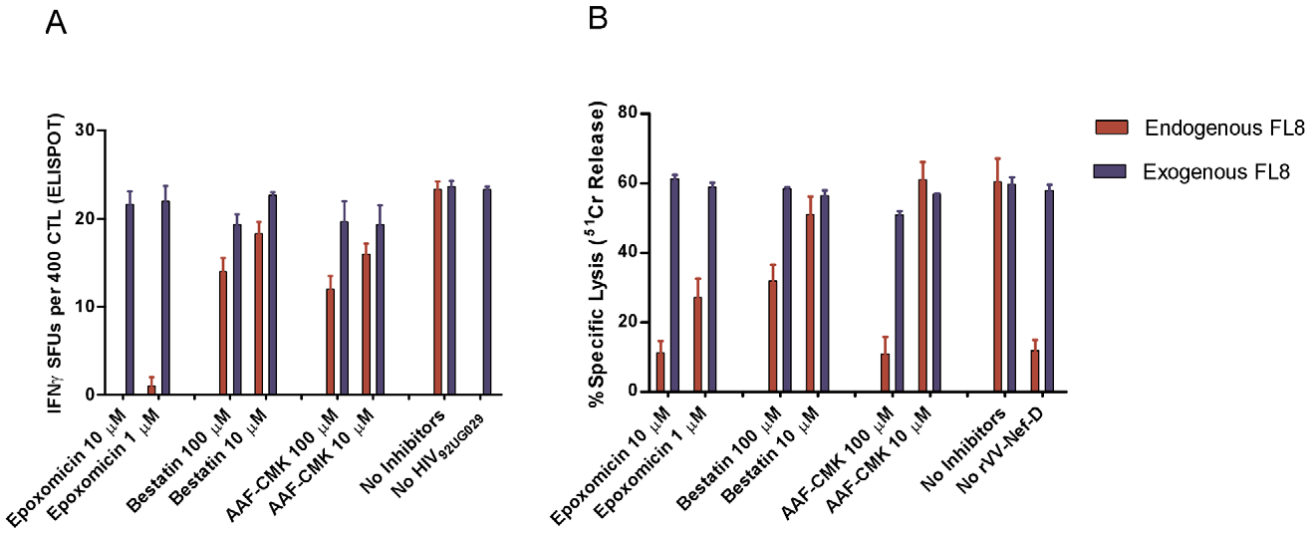
Immunoproteasomes and proteasomes are pivotal to the generation of endogenously processed FL8 epitope. In the intracellular antigen processing inhibition assay (IAPIA), HLA-B8 targets were pre-treated with Epoxomicin (immunoproteasome and proteasome inhibitor), Bestatin (aminopeptidase inhibitor) and AAF-CMK (TPPII protease inhibitor). Target cells were then infected with HIV-1_92UG029_ for 36 hours post-infection and co-cultured with a FL8-specific CTL clone on an IFNy ELISPOT at an E∶T ratio of 400∶20000 per well (A) or infected with rVV-D for 4 hours and then co-cultured with a FL8-specific CTL clone in a chromium release assay at an E∶T ratio of 40000∶5000 per well (B). Red bars represent endogenously derived FL8 peptide for CTL recognition. Blue bars represent exogenously added synthetic FL8 peptide for CTL recognition as an internal control against inhibitor-induced cell death in infected target cells, (with blue bars in the ELISPOT standardised relative to maximum endogenous FL8 SFUs in the absence of inhibitors). The addition of each inhibitor to virally-infected cells was performed in triplicate.

The addition of epoxomicin to high titre HIV-1_92UG029_ infected cells in a modified Live Virus ELISPOT intracellular antigen processing inhibition assay (IAPIA) completely abolished recognition of FL8 at 10 µM (Figure 6A). In contrast, addition of bestatin and AAF-CMK, even at high concentrations of 10–100 µM, had little impact on CTL recognition. A similar pattern was observed when the same three inhibitors were added to BCL infected with rVV-D (HIV-1_94UG114_) in modified chromium release IAPIA (Figure 6B). The addition of epoxomicin at 10 µM and 1 µM reduced CTL lysis by 82% and 53% respectively, whilst addition of Bestatin and AAF-CMK at 10 µM had minimal effect. The marked difference in CTL lysis at 100 µM is likely to represent partial inhibition of proteasomal activity when used at high concentrations. Overall, in contrast to the Nef QK10 epitope [35], the result with AAF-CMK showed that TPPII is not involved in the FL8 processing pathway for the two viruses tested.

Together, these inhibition assays clearly demonstrate that proteasomes and immunoproteasomes play a pivotal role in the endogenous processing of the HLA-B8 FL8 epitope which is required to initiate efficient CTL antiviral activity against HIV-1_92UG029_ and HIV-1_94UG114_ infected targets. Therefore, as a first key step in the processing pathway and in accordance with the antigen processing literature (reviewed in [36], [37]), the cleavage specificities of the immunoproteasomes and proteasomes in particular are likely to have a significant impact on epitope generation correlating with subsequent epitope abundance on the cell surface [38].

### Virus-specific polymorphisms in the FL8 flanking region alter CTL epitope antigen processing

Since the FL8 epitope is conserved between all HIV-1 isolates tested in previous assays, we next investigated whether viral isolate-specific polymorphisms flanking FL8 could modulate the antigen processing efficiency of this epitope. We hypothesised that processing and production of the FL8 epitope may be more efficient in cells infected with clade A isolates HIV-1_92UG029_ and HIV-1_93RW024_, and clade B HIV-1_MN_, whilst impaired antigen processing could account for poor CTL recognition of clade B HIV-1_HXB2_ and HIV-1_89.6_ viruses in Figure 3. Two 25-mer oligopeptides were therefore synthesised to span FL8 (Nef_90–97_) and its flanking region (Nef_82–106_), one corresponding to HIV-1_HXB2_ and the other corresponding to HIV-1_92UG029_. The two oligopeptides differed in the flanking region by 3 amino acids including position 83 (glycine/alanine), 85 (valine/leucine), and 104 (arginine/glutamine). Of these amino acid polymorphisms, the former two can be considered conservative variations, while replacement of arginine by glutamine leads to loss of a basic residue and replacement by an uncharged side chain. To test whether these residues affected antigen processing by the immunoproteasome, which is most commonly involved in CTL epitope generation, each oligopeptide was incubated with purified immuno-20S-proteasome (i20S) for 0, 10, 40 and 70 minutes. The peptide fragments resulting from immunoproteasomal digestion were identified using tandem mass spectrometry. Since immunoproteasomes and proteasomes rarely produce the exact 8–11mer peptide, but instead generate longer epitope pre-cursors that are often correctly cleaved at the C-terminus, we defined an ‘epitope precursor’ as a peptide fragment containing the intact FL8 epitope that is extended at the amino (N) terminus and carboxyl (C) terminus, and a ‘correct epitope pre-cursor’ as a peptide that is extended only at the N-terminus and correctly cleaved at the C-terminus.

**Figure 7:**
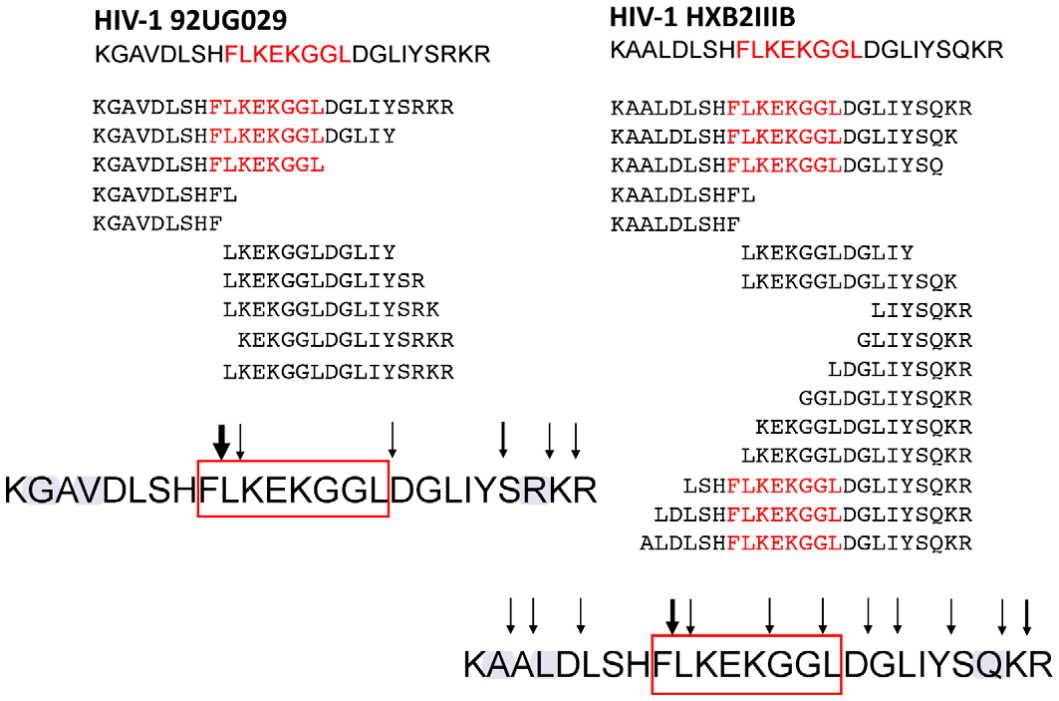
Oligopeptides derived from HIV-1_92UG029_ and HIV-1_HXB2_ showed strikingly different digestion patterns. A pair of 25-mer oligopeptides representing HIV-1_92UG029_ and HIV-1_HXB2_ Nef_82–106_ was incubated with purified immunoproteasome for 0, 10, 40 and 70 minutes and peptide fragments were identified using tandem mass spectrometry. For immunoproteasomal digests analysed at 70 minutes; peptides with an individual ions score >30 are displayed. The cleavage sites within each peptide sequence are indicated by black arrows, with bold arrows indicating frequent cleavage. The conserved FL8 epitope sequence is highlighted in red and virus-specific polymorphisms in the FL8 flanking sequence are highlighted in blue.

**Figure 8:**
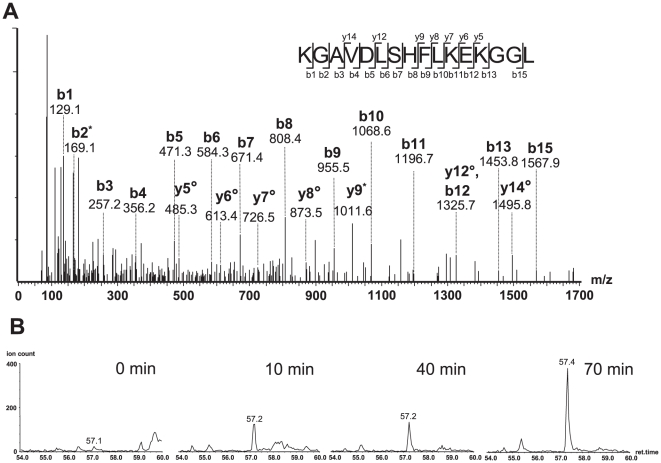
Oligopeptide HIV-1_92UG029_ produced a correct C-terminally cleaved peptide (KGAVDLSHFLKEKGGL). (A) MS/MS spectrum of the peptide KGAVDLSHFLKEKGGL with an observed mass (m/z) of 425.49Da. The assigned b and y fragment ions are indicated. (B) Generation of the peptide KGAVDLSHFLKEKGGL in proteasomal digest. Ion extract chromatograms showing the precursor peptide [M+4H]^4+^ (m/z 425.49Da) demonstrating the accumulation of this proteolysis product over time.

Both oligopeptides showed strikingly different digestion patterns (Figure 7) when digested at these time points. Oligopeptide HIV-1_92UG029_ produced three dominant FL8-containing precursor peptides, of which one was a correct C-terminally cleaved peptide (KGAVDLSHFLKEKGGL) (Figure 8A). Although mass spectrometry is only semi-quantitative, UPLC-MS^E^ analysis at each time point showed that this C-terminally cleaved peptide was present as early as 10 minutes post i20S digestion and increased in quantity at 40 and 70 minutes post digestion (Figure 8B). In contrast, digestions of oligopeptide HIV-1_HXB2_ produced several FL8 containing precursor peptides, but these contained substantial N- and C-terminal extensions, and were only 3 amino acids shorter at most than the original 25-mer oligopeptide. None of the precursor peptides generated from digestion of oligopeptide HIV-1_HXB2_ contained the correct C-terminal cleavage for FL8.

### Critical phenylalanine residues in the N-terminal FL8 flanking region diminish epitope generation

**Figure 9:**
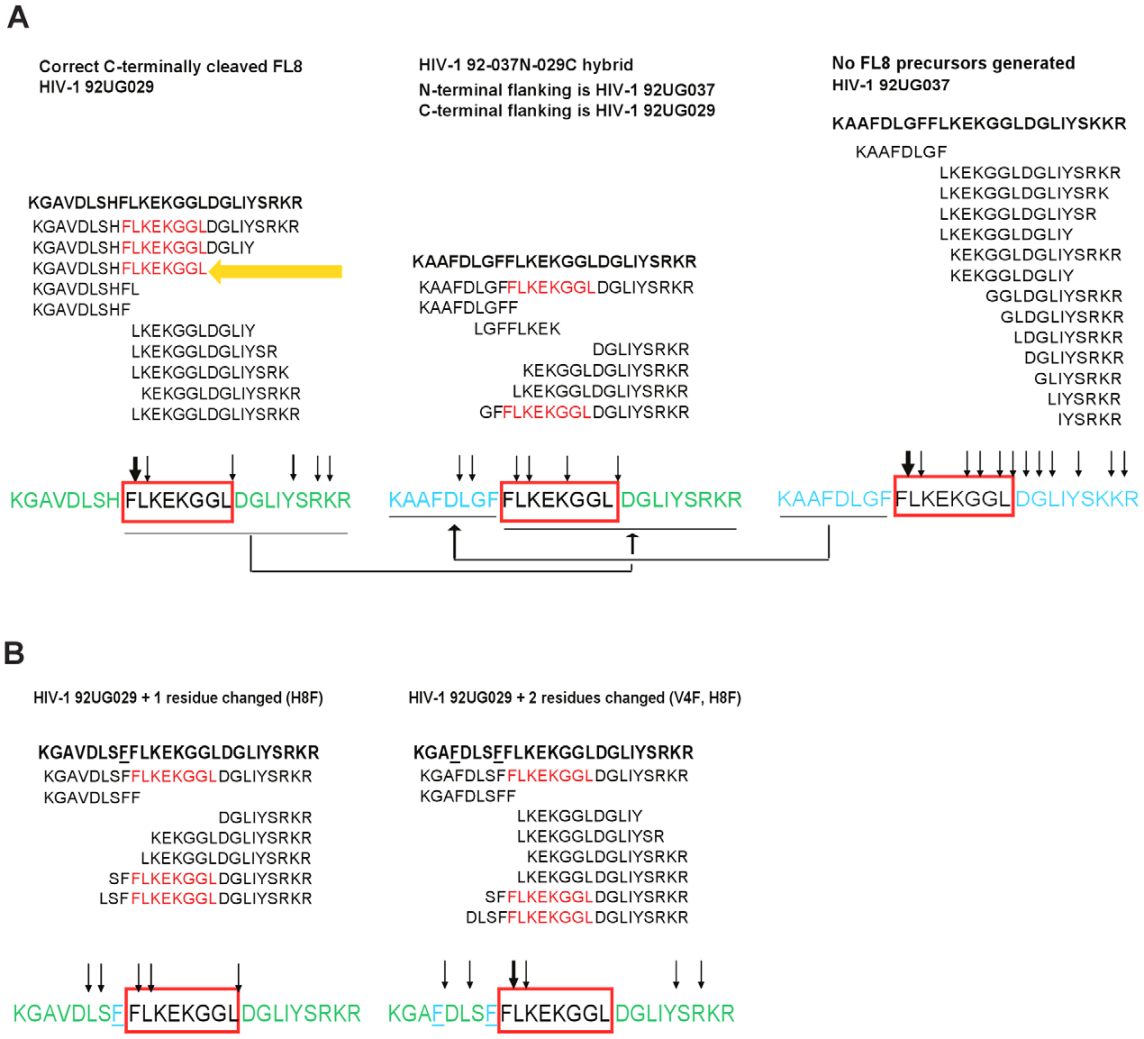
Critical phenylalanine residues in the N-terminal FL8 flanking region diminish epitope generation. (A) Replacing the N-terminal flanking region of HIV-1_92UG029_ with the one of HIV-1_92UG037_ generating a “hybrid” FL8 epitope precursor abolishes the correct C-terminal processing of the FL8 epitope. All three 25-mer oligopeptides representing HIV-1_94UG129_ (left panel), HIV-1_92UG037_ Nef_82–106_ (right panel) and the “hybrid” epitope precursor (middle panel) were incubated with purified immunoproteasome for 0, 10, 40 and 70 minutes, and the resulting peptide fragments were identified using tandem mass spectrometry. Peptide fragments with an individual ions score >30 are shown for immunoproteasomal digests analysed at 70 minutes. The cleavage sites within each peptide sequence are indicated by black arrows, with bold arrows indicating frequent cleavage. The conserved FL8 epitope sequence is highlighted in red and virus-specific polymorphisms in the FL8 flanking region are highlighted in blue. (B) Phenylalanine substitutions at position 89 and 85 in HIV-1_92UG029_ abolished C-terminal processing of the FL8 epitope. MS based analysis of resulting peptide fragments were performed as described in (A).

Because of the large number of viral isolate-specific polymorphisms in the different viruses tested, it is difficult to define accurately which amino acid polymorphism(s) in the flanking regions present in different HIV-1 isolates critically impair immunoproteasomal cleavage of FL8. We speculated that one or several amino acid polymorphisms may act in tandem to alter cleavage patterns. Since most of the flanking amino acid differences were in the N-terminal part of the FL8 epitope, we “swapped” the N-terminal sequence of the FL8 epitope region from the HIV-1_92UG029_ virus that is recognized by CTL with the sequence derived from the rVV-Nef-A(HIV-1_92UG037_) virus that is not recognized (see Table 1 and Figure 9A) to create a “hybrid” FL8 epitope precursor. When digested by proteasomal proteolysis and analysed by UPLC-MS^E^, no correct C-terminal cleavage was observed, indicating that the N-terminal region of the FL8 epitope is critical for this cleavage step. We therefore further examined the polymorphisms observed in the PCR-derived sequence ‘tracked’ to either superior or abolished antiviral efficacy in our assays (Table 1). Interestingly, polymorphisms in our live viral sequences did not track to CTL antiviral efficacy. Conversely, we noted that the presence of a phenylalanine at position 89 immediately adjacent to the N-terminus of the FL8 epitope and an additional phenylalanine at position 85, correlated with abolished CTL antiviral efficacy in three rVV isolates; A, AE and G. Variation of N- and C-terminal epitope flanking residues can influence the length and nature of epitope precursors that are generated [39] [40] [41] and the immunoproteasome is known to have a preference for cleaving after hydrophobic residues [42]. Thus, this motif is likely to have a pronounced impact on FL8 cleavage patterns. We therefore repeated the *in vitro* proteasomal digestion assay to test whether this phenylalanine motif may play a key role in modulating epitope processing. We chose two previously studied viruses that exhibited contrasting epitope generation in digests (associated with contrasting CTL antiviral efficacy); HIV-1_92UG029_ and rVV-Nef-A(HIV-1_92UG037_). Previous digestion of the HIV-1_92UG029_ derived precursor peptide generated a correct C-terminally cleaved FL8 pre-cursor that was present in large quantities. In contrast, digestion of the HIV-1_92UG037_ derived precursor peptide containing this phenylalanine motif generated no FL8-containing pre-cursors at any of the time points tested (Figure 8). Therefore, we took the HIV-1_92UG029_ sequence (KGAVDLSH**FLKEKGGL**DGLIYSRKR) and designed two new 25-mer oligopeptides; the first in which we substituted the basic histidine residue with a large hydrophobic phenylalanine at position 89 immediately adjacent to FL8 (HIV-1_92UG029_+1: KGAVDLSF**FLKEKGGL**DGLIYSRKR) and an additional substitution at position 85 (HIV-1_92UG029_+2: KGAFDLSF**FLKEKGGL**DGLIYSRKR) in which hydrophobic valine was replaced with a hydrophobic phenylalanine. The original oligopeptide and two new oligopeptides were then digested with immunoproteasome for 0, 10, 40, and 70 minutes and peptide fragments were identified using tandem mass spectrometry (Figure 9). The insertion of the phenylalanine motif markedly altered the pattern of immunoproteasomal cleavage. Whilst the digestion of the original HIV-1_92UG029_ sequence generated the correct C-terminally cleaved FL8 pre-cursor in high quantity, the +1 and +2 oligopeptide sequences did not generate any C-terminally cleaved pre-cursor peptides. For the +1 oligopeptide, the breadth of intra-epitope cleavage was enhanced in comparison to the original peptide (in which intra-epitope cleavage site was predominantly focused after the F). For the +2 oligopeptide, despite repeat analyses, only three fragments were identified in addition to the mother peptide. This is indicative that the motif markedly alters both cleavage patterns and the overall quantity of pre-cursors generated. Collectively, these data demonstrate that the phenylalanine motif in the N-terminal sequence can diminish epitope processing, and highlight that even a single virus-isolate polymorphism (H89P) in the flanking region can substantially alter epitope production.

## Discussion

We have shown that striking differences exist in CTL antiviral efficacy to a conserved epitope shared between diverse viral isolates, and that from the panel of HIV-1 isolates only 23% were recognized by CTL. In experiments with five high titre HIV-laboratory strains *in vitro*, FL8-specific CTL demonstrated efficient viral suppression and a strong IFNγ response against endogenous FL8 peptide presented by cells infected with clade isolate HIV-1_93RW024_, and clade B HIV-1_MN_, and sub-optimal antiviral activity to clade A isolate HIV-1_92UG029_. However, no CTL antiviral activity was detected against a further two clade B strains, HIV-1_HXB2_ and HIV-1_89.6_. We also utilised a recombinant vaccinia virus system expressing Nef from eight viral isolates, each from a different group M clade (A–H), to evaluate whether FL8 epitope-specific CTL recognition and antiviral activity differs between viral isolates. Again, we observed significant differences in CTL lysis and IFNγ secretion, with correct epitope processing and CTL recognition completely abolished in three viral isolates (A, AE and G) and impaired in a further four isolates (B, C, F, H). Collectively, we demonstrate that a surprisingly large proportion (77%) of Nef proteins with a conserved FL8 epitope, expressed by HIV-1 isolates or as recombinant vaccinia-Nef were suboptimally recognised by FL8- specific T-cells, despite the presence of the same epitope. Both endogenous expression systems clearly show that the antiviral efficacy of CD8+ T cells to an invariant epitope is heavily dependent on the infecting viral isolate, which occurs independently of clade-grouping. We have shown that inter-clade and intra-clade polymorphisms in the FL8 flanking region modulate epitope processing by the immunoproteasome, to enhance or impair epitope generation, which is associated with altered CTL recognition and antiviral activity in the infecting HIV-1 strains tested. Furthermore, we demonstrate that the ‘swapping’ of the flanking regions between viruses that are recognised and not recognised can appreciably modulate epitope processing, and we identify a N-terminal phenylalanine motif that can diminish epitope generation. When modified, this can help to optimize CTL responses elicited by appropriately designed vaccine vectors containing critical flanking sequences in addition to the epitope that enhance epitope antigenicity.

Although it has been previously shown that flanking residues can impact epitope presentation by MHC, also for HIV-1 derived epitopes [19], [23] such studies have focused on identifying amino acid variation predominantly within a single viral isolate. In contrast, our study evaluated the reason for variable CTL responses despite absolute conservation of a HIV Nef epitope shared by a panel of 13 HIV-1 whole viral isolates and recombinant vaccinia-Nef. Strikingly, our results strongly suggest that the impact of viral variation on efficient antigen processing has been seriously underestimated, and this has been reinforced by a reliance on peptide-based assays to measure T-cell responses in natural history and vaccine studies. Due to the current focus on developing HIV vaccines that elicit T-cells to conserved regions of the viral genome, this is an important finding that has implications for both vaccine design and evaluation of vaccine efficacy.

Our results emphasise that the antiviral efficacy and cross-reactive potential of CD8+ T-cells should be assessed by their ability against cells infected with virus, and it cannot be accurately predicted solely on the basis of epitope conservation or based on the results of CTL assays using exogenously-loaded peptides. Since infecting viral isolates can exploit impaired antigen processing and presentation to hide from immune surveillance, epitope conservation between viruses may not accurately predict the cross-reactive potential and antiviral efficacy of CTL. The lack of epitope presentation in particular viral backbones may be one of the reasons that high levels of CTL elicited in people who become super-infected, either after stable HIV-1 infection [43] or after vaccination [44], appear to be ineffective even when the infecting virus contains the identical epitope. Our data suggest that potential immunogens for cross-clade vaccine design should not be based solely upon invariant epitopes, but should focus upon conserved regions that include similar epitope flanking regions which have been tested via functional assays for endogenous presentation prior to use in vaccine constructs. Similarly, it will be important to measure the ability of vaccine elicited T cells to recognize a range of different virus isolates in whole-virus assays as well as the more conventional peptide-based studies. The use of whole-virus assays is not only informative on the efficacy of antigen processing and presentation, but can also be adapted to detect additional changes in CTL antiviral efficacy attributable to viral functions; which include differential protein expression kinetics, variation in protein expression levels by Tat, and the down-regulation of MHC class I by Nef [31]. For future vaccine design, it may also be necessary to reassess the relative value of utilising particular highly conserved and immunogenic regions of the virus, such as this FL8 epitope or the Gag p24 region [10], [45], since sequence conservation may mask a lack of presentation that undermines the efficacy of vaccine-induced responses. Interestingly, since Gag is much less variable between HIV-1 primary isolates and also during the natural course of infection due to fitness costs associated with changes in sequence [46], the probability of gag epitopes being processed more efficiently among diverse viral isolates may be higher than for epitopes from other HIV proteins. This could potentially contribute to the efficacy of Gag-specific CTL observed in chronic HIV-infected patients [47].

Inter-clade and intra-clade virus specific polymorphims may also shape immunodominance as the targeting of HIV-specific CTL epitopes in a hierarchical pattern is sensitive to alterations in antigen processing and presentation [48]. FL8 is a well characterised immunodominant epitope during acute infection, with FL8-specific T-cells detected in over 70% of HIV-infected HLA-B8 participants tested [49]. Yet, immunodominance does not necessarily mean that the FL8 epitope is optimally processed in the majority of viral strains within each infected individual. The low antigen load generated by a viral isolate with sub-optimal or impaired epitope processing may be sufficient to prime T-cells, but insufficient to trigger cytotoxic killing *in vivo*, especially by low avidity CTL. In addition to the impact of viral isolate-specific polymorphisms, the high mutation rate of HIV may give rise to potential intra-epitopic and epitope-flanking escape mutations during acute infection [50] that subsequently diminish processing. Therefore, the T cells primed by the infecting virus might not be able to control mutated viruses that emerge during the course of infection. Also, strong immune pressure upon neighbouring epitopes such as B57-KF9 Nef in the first few weeks post-infection [13] or on epitopes overlapping with FL8, such as B60-KL9 [51], A2-FL11, A3-AK9, A3-DK9, A24-HL9, may alter the generation and consequent immunogenicity of FL8. Furthermore, in the absence of sufficient antigen, where cells are infected by non-infectious virus or epitope processing is abolished, dendritic cells may acquire these HIV-infected targets and successfully cross-present HIV antigens to prime the expansion of HIV-specific T-cells [52]. Collectively, these mechanisms may explain why FL8-specific T-cells are immunodominant in acute infection, but unlike other responses, FL8 is an outlier that does not correlate with viral control at set point [53].

In our study, we have used rVV in addition to whole HIV to demonstrate our major conclusion that the anti-viral efficacy of B8 FL8 restricted responses are heavily dependent on viral isolates. Since the backbones for rVV and whole virus are different, it is possibile that rVV may not fully represent whole virus, and vaccinia expression levels and HIV expression levels are not equivalent. Ideally, direct HIV to HIV comparison would be very useful for the hypothesis. However, it is worth noting that the ELISPOT by using live virus (MN) infected cells or Vaccinia virus(MN) both give detectable FL8-specific responses which is indicative that the two assays are comparable. Processing data by using the sequences derived from rVV further confirmed the initial observation utilising rVV in IFNγ ELISPOT and lytic assays. Interestingly, analysis of viral amino acid sequence (arising from PCR amplification and sequencing of our infected targets) consistently differed from the expected Los Alamos National Laboratory (LANL) HIV Sequence Database for each of the five viruses, although the FL8 epitope remained unchanged. Whilst some of the viruses used in our assays were molecular clones, others were clinical isolate swarms, and therefore we could not exclude the possibility that there is heterogeneity in the FL8 epitope or flanking sequences. Yet consistent sequencing results were obtained when multiple PCR were performed, suggesting sequences showed in table 1 were dominant sequences. Considering that HIV undergoes an average of 1 mutation per genome per replicative cycle due to the error-prone nature of the reverse transcriptase, some mutations may become ‘fixed’ or continue to evolve in *in vitro* assays in which CD4+ T-cells are infected with HIV-1 laboratory strains, even under no selection pressure. Due to this high mutation rate, the LANL database represents a ‘snap shot’ of viral genomes and proteomes at a single time point, and therefore repeated sequencing is necessary.

Our *in vitro* digests utilised immunoproteasomes as they are typically induced within the first day of viral infection by exposure to IFNγ or TNFα [54] [55]. Although immunoproteasomes can generate a different spectrum of epitopes from standard 20S proteasomes, the effects are thought to be relatively subtle, with more pronounced quantitative rather than qualitative differences [56]. Current estimates suggest that only 15% of peptides are the appropriate 8–11mer length for MHC-loading after proteasomal or immunoproteasomal digestion [57], therefore the generation of extended FL8 precursors is not unusual. The N-terminal extensions are usually trimmed by aminopeptidases in the cytosol or after TAP-mediated transfer into the ER [58], although successful CTL recognition of an endogenously derived 15-mer N-terminally-extended HIV-1 gp160 peptide was recently observed [59]. Since more than 99% of peptides are thought to be destroyed by cytosolic peptidases before encountering TAP [60], a limitation to this technique is that the liberation of FL8-containing precursors in our *in vitro* assays cannot guarantee efficiency in subsequent processing and presentation steps of FL8. However, the importance of the proteasome in liberating Nef peptides, and the strong correlation between the identities of extended epitopic precursors generated via *in vitro* proteasomal cleavage and naturally processed peptides acid-eluted from the surface of Nef-transfected cells, indicate that *in vitro* digests are a reliable tool [38], [61]. These studies on Nef, together with our *in vitro* inhibition assays and immunoproteasomal digestions, strongly support the central role of the proteasome and immunoproteasome in altering peptide generation. In addition, since the specificity of TAP transport, ERAAP trimming and also HLA-binding affinity are dictated by internal epitope composition, particularly the C-terminal residue [37], they are likely to be similar between viral strains with a conserved epitope (post-proteasomal proteolysis). Therefore, these steps further along in the processing pathway are expected to have limited influence on the generation of the conserved FL8 epitope studied here, although this warrants further investigation. Interestingly, the overlapping HLA-B*60 KL9 epitope (KEKGGLEGL) is flanked by the amino acids FL at the N-terminus [49], and the FL8 epitope (FLKEKGGL) is flanked by SH. These differences can be sufficient to determine a completely different outcome in their processing efficiency by the proteasome.

In conclusion, our findings show that striking differences exist in the antiviral efficacy of CTL to an invariant epitope shared between viral isolates. Only a small proportion (23%) of the HIV-1 Nef proteins, expressed by HIV-1 isolates or as recombinant vaccinia-Nef, elicited optimal FL8-specific CTL antiviral responses, whilst the majority demonstrated impaired or completely abolished epitope processing. We found that virus-specific polymorphisms in the flanking region to a conserved epitope substantially alters immunoproteasomal proteolysis, favouring, impairing or completely abolishing epitope generation, which is correlated with the efficiency of CTL antiviral activity *in vitro* and may play an important role in determining epitope immunogenicity and immunodominance required to prime T-cells *in vivo*. CTL antiviral efficacy is heavily dependent on the infecting viral strain; this occurs independently of clade-grouping. This is of major relevance for the design of future vaccines protective against genetically diverse strains of HIV-1, and should be carefully evaluated when assessing the effectiveness of vaccine-induced T-cell responses.

## Materials and Methods

### Viruses

HIV-1 viral isolates 92UG029, 93RW024 (clade A) were obtained from The UNAIDS Network for HIV Isolation and Characterization, and the DAIDS, NIAID. HIV-1 laboratory strains IIIB, MN, 89.6 (clade B) were obtained from the AIDS Research and Reference Reagent Program, Division of AIDS, NIAID, NIH, in addition to eight recombinant vaccinia viruses expressing Nef protein from different group M clades (specified within brackets): HIV-1_92UG037.1(A),_ HIV-1_MN_ (B), HIV-1_96ZM651_ (C), HIV-1_94UG114.1_ (D), HIV-1_CM235-32_ (AE), HIV-1_93BR020_ (F), HIV-1_92NG83.2_ (G) and HIV-1_90CF056_ (H). Details and acknowledgment of each reagent are listed in supplemental material.

### CTL clones and HIV-1 permissive target cells

CTL clones specific for HLA-B8 epitopes were generated by limiting dilution from the PBMCs of HIV-infected patients responding to the FL8 and EI8 epitope and maintained as described in Dong et al 2004 [62]. The Human T cell leukaemia C8166 line stably expressing CD4+ and HLA-B8 was maintained in R10 media (RPMI 1640 with 100 U/ml of penicillin, 100 U/ml of streptomycin, 2 mM L-glutamine and 10% heat-inactivated fetal calf serum from Sigma-Aldrich).

### CTL lysis assays

CTL lysis assays were performed using standard ^51^Chromium release assays as described elsewhere [63]. Slight modifications were made to test infection with recombinant vaccinia viruses (rVV). In brief, a B-cell line (BCL) expressing HLA-B8 was incubated with 150 µCi of chromium for one hour at 37°C/5% CO_2_, then washed extensively prior to infection with rVV encoding HIV-nef genes at 2×10^6^ pfu/million cells for one hour in serum-free RPMI. Infected cells were allowed to recover for a further two hours in R10. The labeled target cells were transferred to a round bottom 96-well plate and co-cultured with HLA-matched CTL clones in triplicate at an E∶T ratio of 40000∶5000 per well for a further 4 hours at 37°C. Supernatants were harvested and radioactivity was assessed using a beta-plate counter (Wallac). Spontaneous chromium release was determined by analysing the release from target cells incubated in R10 media and maximum release of incorporated chromium was obtained from target cells treated with 5% Triton X-100 detergent. Specific lysis was calculated = 100 × (experimental lysis − spontaneous lysis)/(maximum lysis − spontaneous lysis).

### Human IFNγ Elispot assay

Standard Human IFNγ ELISPOT assays were performed as described elsewhere [62]. Slight modifications were made when testing recombinant vaccinia viruses (rVV). In brief, a HLA-B08 expressing BCL was infected with rVV encoding HIV-nef genes at 2×10^6^ pfu/million cells for one hour in 1 ml RPMI and recovered as described above. Subsequently, the infected target cells were washed twice and added to pre-coated IFNγ ELISPOT plates with a HLA-matched CTL clone at an E∶T ratio of 400∶20,000, in triplicate, in a final volume of 100 µ/well. Control wells included uninfected BCL (negative) and peptide pulsed uninfected BCL (positive) plus peptide pulsed rVV-infected BCL co-cultured with CTL. Assays were incubated for 6 hours at 37°C/5% CO_2_ and developed as normal.

### Live virus ELISPOT (LVE)

Differing HIV-1 strains (2-fold TCID_50_) were used to infect C8166 cells. Infected cells were washed twice, split with at least 1×10^6^ cells/T25 flask cultured in a total volume of 2 ml R10 for a period of 6, 24, 48, 72 and 96 hours post-infection at 37°C/5% CO_2_. At each time point, cells were washed, counted and co-cultured with the panel of HLA-matched CTL clones in triplicate at one E∶T ratio of 400∶20000 on the pre-coated interferon gamma IFNγ ELISPOT plates at a final volume of 100 µl/well. Negative controls included the individual CTL clones co-cultured with uninfected target cell line in triplicate, and positive control included each CTL clone co-cultured with uninfected target cells pulsed with 2 µM of specific peptide. ELISPOT plates were incubated for 6 hrs at 37°C/5% CO_2_ and subsequently washed and developed as described previously. Spot forming units (SFUs) were counted using the ELISPOT reader system AID ELIspot 4.0.

### Viral suppression assay (VSA)

Differing high titre HIV-1 strains (TCID_50_) were used to infect the C8166 cell pellets during a 90 minute incubation at 37°C/5% CO_2_ and were subsequently washed (2×) to remove free virus. 5×10^4^ infected cells were co-cultured with HLA-matched HIV-1 specific CTL clones at differing E∶T ratios of 1∶1, 1∶2, 1∶4, 1∶8 & 1∶16 in H10-IL2 (200 U/ml) on a flat bottom 96-well plate, in a final volume of 200 µl per well, at 37°C for 4 days. Each condition was performed in triplicate, including one HLA-mismatched clone as a negative control and virus-infected cells in the absence of CTL as a positive control. Suppression of infected cells by CTL on Day 4 was directly monitored using an intracellular anti-p24 gag mAb (KC57-RD1, Beckman Coulter). The extracellular p24 content in the supernatant was also assayed by quantitative p24 antigen ELISA (Immunodiagnostics) in accordance with the manufacturer's protocol.

### Proviral DNA isolation and sequencing

Proviral DNA was isolated for each high titre virus from control wells containing only HIV-infected C8166 cells in the viral suppression assay using the PureGene DNA isolation Kit (Gentra Systems) as per the manufacturer's instructions. Nef was amplified from proviral DNA by nested PCR. The nested PCR amplification was carried out in a total volume of 50 µl using the Taq polymerase PCR reaction kit (QIAGEN) and AccuPrime Taq DNA Polymerase High Fidelity kit (Invitrogen), according to the manufacturer's instructions. GTA GCT GAG GGG ACA GAT AG and TGC TAG AGA TTT TCC ACA C. Initial denaturing at 94°C for 120 seconds was followed by 30 cycles of denaturing at 94°C for 30 seconds, annealing at 52°C for 30 seconds and extension at 72°C for 90 seconds. A final extension at 72°C was run for 300 seconds. The internal pair of primers used was GAA GAA TAA GAC AGG GCT and AGG CTC AGA TCT GGT CTA A. Initial denaturing at 94°C for 120 seconds was followed by 30 cycles of denaturing at 94°C for 30 seconds, annealing at 56°C for 30 seconds and extension at 72°C for 90 seconds, with a final extension at 72°C was run for 300 seconds. The PCR products were checked for size on a 1% agarose gel and sequencing was performed in the MRC HIU Sequencing Facility, Weatherall Institute of Molecular Medicine.

### Flow cytometry for HLA-B8 surface expression

1 million C8166 were infected separately with the five high titre viruses (TCID_50_) for 48 hours whilst 1 million BCL were infected separately with the eight rVV (2×10^6^ pfu/million targets) for four hours. Both sets of virus-infected cells were stained with a biotin-conjugated anti-HLA-Class I B8 monoclonal antibody (AB33716, Abcam) washed twice, and fixed with 1% paraformaldehyde. HLA-B8^−^ negative cells were used to set the negative quadrants and to confirm negligible cross-reactivity of the antibody to other HLA-alleles expressed on target cells. Additional controls included the biotin-conjugated antibody in the presence and absence of streptavidin and uninfected cells. At least 10^5^ live cells per infection were counted using a Cyan flow cytometer and analysed with FlowJo.

### Intracellular antigen processing inhibition assays (IAPIA)

The C8166 cells were pre-treated with proteasome inhibitor Epoxomicin, TPP II inhibitor AAF-CMK and aminopeptidase inhibitor Bestatin (all Biomol) at varying concentrations at 37°C for one hour prior. The cells were then infected with HIV-1_92UG029_ at TCID_50_ for 90 minutes at 37°C/5% CO_2_. Two controls comprising pre-treated cells not infected with HIV (to determine the affect of the inhibitor on cell survival in the absence of HIV-induced cell death), and HIV infected untreated cells (to determine the maximum response elicited in the absence of inhibitor treatment) were used. All cells were washed after 90 minutes and transferred to a T25 flask containing R10 medium for 36 hrs before being used as targets in a modified Live Virus Elispot with a FL8-specific CTL clone at an E∶T ratio of 400∶20000 as described previously. Slight modifications were made to test infection with recombinant vaccinia virus (rVV) expressing Nef from HIV-1 clade D isolate HIV-1_94UG114_. In brief, BCL expressing HLA-B08 was incubated with 150 µCi of chromium for one hour at 37°C/5% CO_2_ and then washed extensively. The BCL were pre-treated with proteasome inhibitor Epoxomicin, TPPII inhibitor AAF-CMK and aminopeptidase inhibitor Bestatin (all Biomol) at varying concentrations in serum-free RPMI for one hour at 37°C. The cells were then infected with rVV-Nef-D at 2×10^6^ pfu/million targets for one hour in serum-free RPMI, before recovery in R10 for a further one hour. The labelled target cells were then transferred to a round bottom 96-well plate and co-cultured with a HLA-matched CTL clone in triplicate at an E∶T ratio of 40000∶5000 per well for a further 4 hours at 37°C. Supernatants were harvested and radioactivity was assessed as described previously.

### Proteasomal digestion assay (PDA)

Four 25-mer oligopeptides were designed to cover the FL8 epitope and flanking regions corresponding to the amino acid sequence as determined by sequencing for HIV-1_HXB2IIIB_, HIV-1_92UG029_ and HIV-1_92UG037_ and hybrid viral sequences. Extended peptides

KAALDLSH**FLKEKGGL**DGLIYSQKR (HIV-1_HXB2IIIB_), KGAVDLSH**FLKEKGGL**DGLIYSRKR (HIV-1_92UG029_), KAAFDLGF**FLKEKGGL**DGLIYSKKR (HIV-1_92UG037_), KAAFDLGF**FLKEKGGL**DGLIYSRKR (HIV-1_92037N-029C_), KGAVDLSF**FLKEKGGL**DGLIYSRKR) (HIV-1_92UG029+1_), KGAFDLSF**FLKEKGGL**DGLIYSRKR) (HIV-1_92UG029+2_): containing the HLA-B8 restricted FL8 epitope FLKEKGGL (indicated in bold) were synthesized by solid-phase F-moc chemistry on an automated peptide synthesizer (Advanced ChemTech) and purified to >98% by reversed-phase HPLC. *In vitro* proteasomal digestion of synthetic 25mer oligopeptides was conducted using immuno-20S (i20S) proteasome purchased from Biomol International, essentially as described [64]. For each oligopeptide, 1 µg of immuno-20S proteasome was incubated with 10 µg of peptide in a final volume of 100 µL of 20 mM Hepes, pH 7.8, 2 mM magnesium acetate and 2 mM dithiothreitol and incubated at 37°C/5% CO_2_. Sample aliquots were taken at several time points (0, 10, 40, 70 minutes) and reactions were terminated by the addition of 0.1 volume of formic acid (FA). Control reactions without the i20S-proteasome were analysed to evaluate non-specific peptide degradation. The i20S-proteasome was pelleted by ultracentrifugation at 100000 g for 5 hours, and supernatants containing digested peptides were analysed using nano UPLC-high/low collision energy switching MS (MS^E^) as described [65]. In brief, the peptide digests were subjected to chromatographic separation by a NanoAcquity UPLC system coupled to a QTof premier tandem mass spectrometer (Waters, Milford, MA, USA). For peptide precursor and fragment identification and simultaneous quantification, the instrument was run in MS^E^ mode. Peptide peaks corresponding to the original undigested and shorter peptide fragments resulting from immunoproteasomal digestion were identified using a MASCOT search engine (version 2.2) and a custom made database containing HIV-peptide sequences. Quantitative information was obtained from extracted ion chromatograms using MassLynx 4.1 software.

## Supporting Information

Figure S1 The viruses utilized in our assays are not Nef deficient and Nef expression levels are similar. High titre viruses HIV-1HXB2 (A), MN (B) and 92RW024 (C) have a conserved Nef A24-RW8 epitope that induces strong RW8-specific CTL responses in Live Virus Elispots. Recombinant vaccinia-viruses rVV-A, -AE and -G have a conserved Nef A3-QK10 epitope that elicits CTL antiviral activity in a lytic chromium release assay (D). HIV-189.6 and UG029 exhibited a defined RW8 intra-epitopic variant at position two (2F) that is known to result in the loss of CTL recognition via altered epitope processing.
(0.32 MB TIF)

Figure S2 Similar results are observed between the use our HLA-B8 cell line and primary HLA-B8 CD4+ T-cells when target-cells were infected separately with HIV-MN (the FL8 epitope was processed) and HIV-IIIB (the FL8 epitope wasn't processed). Primary CD4+ T-cells expressing HLA-B8/B7 were infected separately with HIVHXB2IIIB and HIVMN in a viral suppression assay (p24 Elisa) with CTL clones specific for the HLA-B8 Nef FL8 epitope and also CTL clones specific for a HLA-B7 Nef RL10 epitope as an internal control.
(1.28 MB TIF)
