# Supplementary figures and images for: The Antiviral Efficacy of HIV-Specific CD8+ T-Cells to a Conserved Epitope Is Heavily Dependent on the Infecting HIV-1 Isolate
Source: PLoS Pathog. 2011 May 12;7(5):e1001341. doi: 10.1371/journal.ppat.1001341 (PMC3093356; doi:10.1371/journal.ppat.1001341)

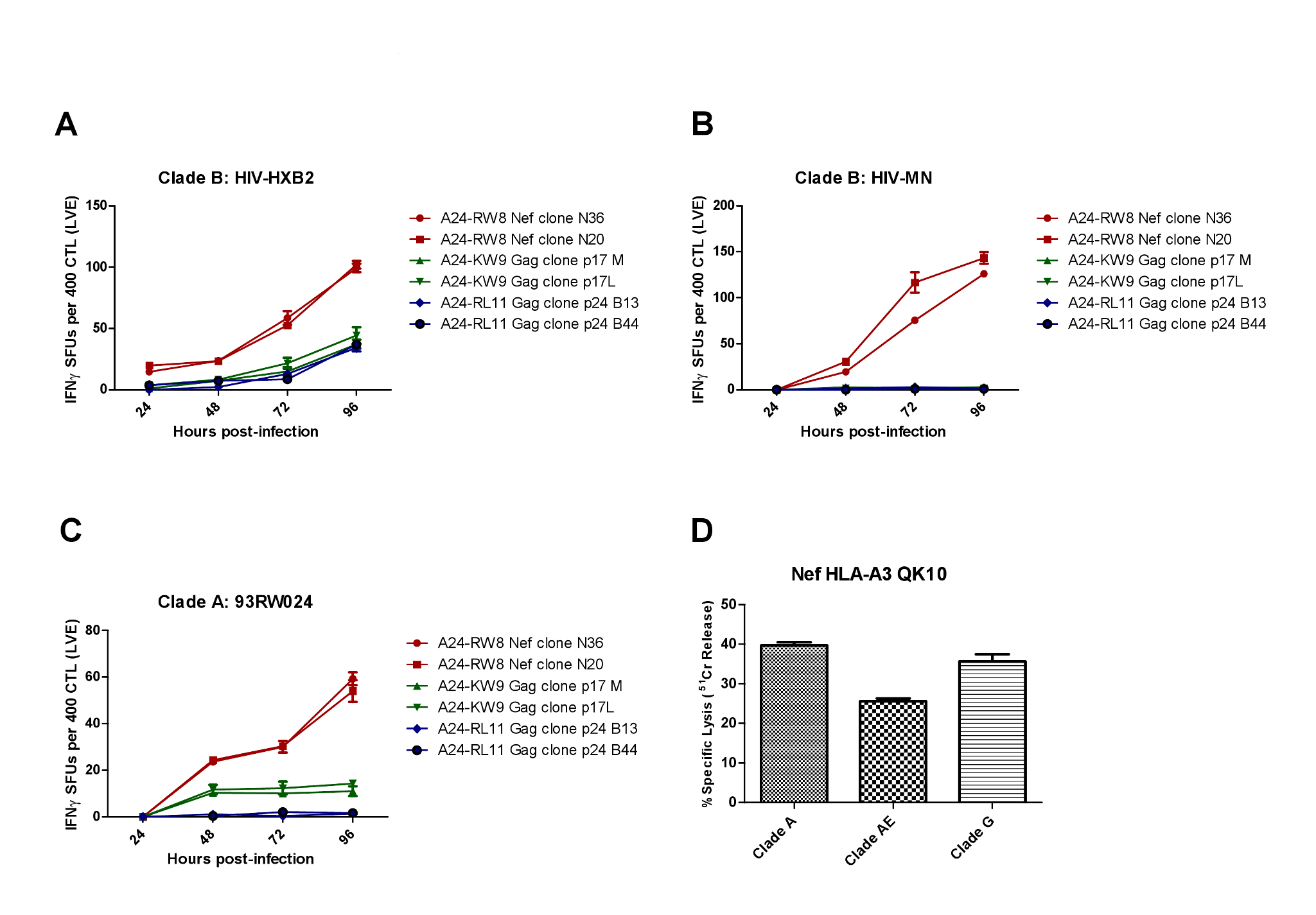

Supplement: Figure S1 — The viruses utilized in our assays are not Nef deficient and Nef expression levels are similar. High titre viruses HIV-1HXB2 (A), MN (B) and 92RW024 (C) have a conserved Nef A24-RW8 epitope that induces strong RW8-specific CTL responses in Live Virus Elispots. Recombinant vaccinia-viruses rVV-A, -AE and -G have a conserved Nef A3-QK10 epitope that elicits CTL antiviral activity in a lytic chromium release assay (D). HIV-189.6 and UG029 exhibited a defined RW8 intra-epitopic variant at position two (2F) that is known to result in the loss of CTL recognition via altered epitope processing. (0.32 MB TIF) [file ppat.1001341.s001.tif]

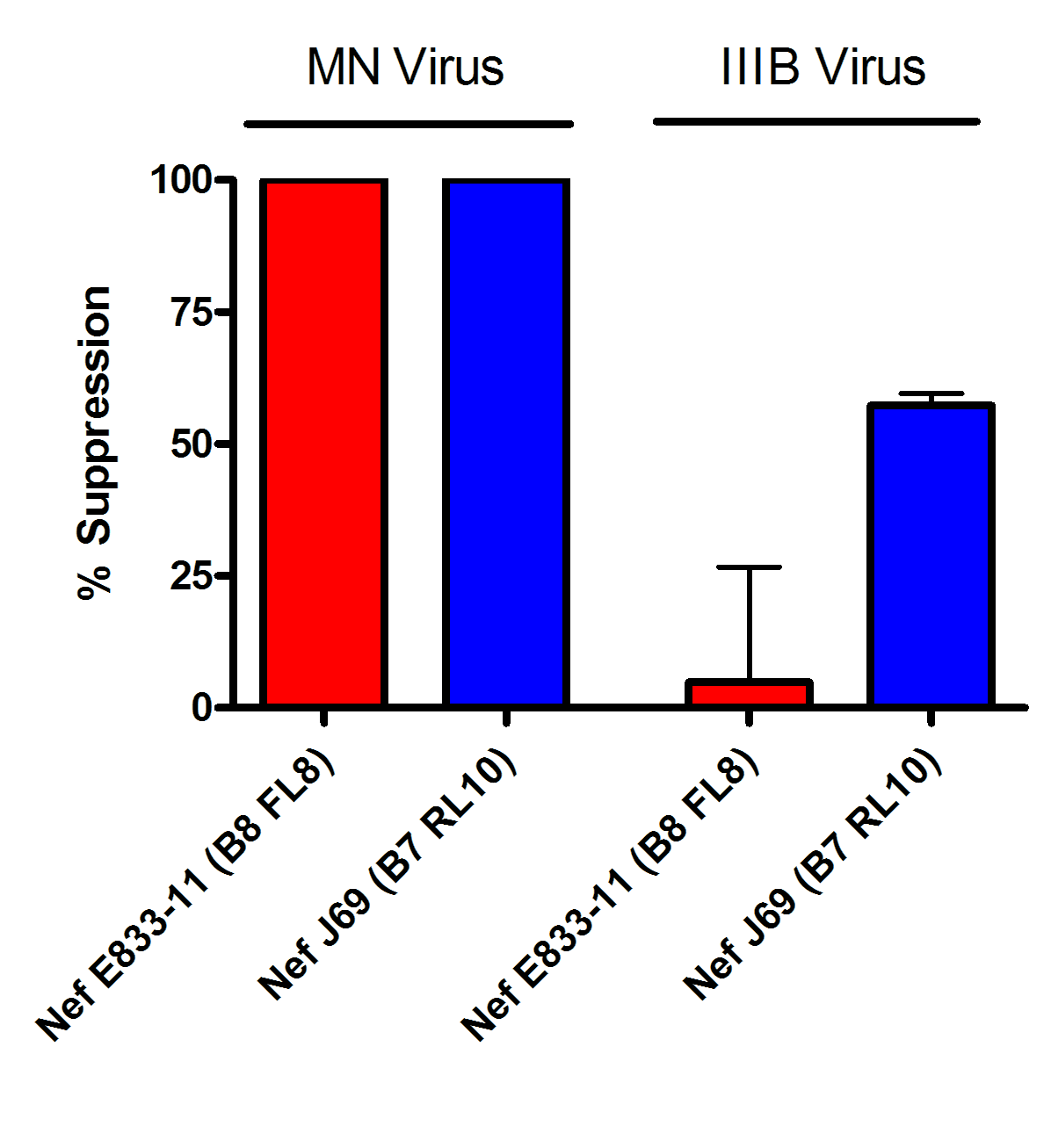

Supplement: Figure S2 — Similar results are observed between the use our HLA-B8 cell line and primary HLA-B8 CD4+ T-cells when target-cells were infected separately with HIV-MN (the FL8 epitope was processed) and HIV-IIIB (the FL8 epitope wasn't processed). Primary CD4+ T-cells expressing HLA-B8/B7 were infected separately with HIVHXB2IIIB and HIVMN in a viral suppression assay (p24 Elisa) with CTL clones specific for the HLA-B8 Nef FL8 epitope and also CTL clones specific for a HLA-B7 Nef RL10 epitope as an internal control. (1.28 MB TIF) [file ppat.1001341.s002.tif]
